# Evaluation of hyaluronic acid gel dissolution with hyaluronidase in an in-vitro prostate cancer model

**DOI:** 10.1016/j.ctro.2021.12.009

**Published:** 2022-01-05

**Authors:** Ben G.L. Vanneste, Ludy Lutgens, Evert J. Van Limbergen

**Affiliations:** Department of Radiation Oncology (MAASTRO), GROW - School for Oncology and Developmental Biology, Maastricht University Medical Center+, Maastricht, the Netherlands

**Keywords:** Rectum spacer, Hyaluronic acid, Hyaluronidase, In-vitro, Prostate cancer radiotherapy

## Abstract

**Aim:**

To determine a dose response relationship of disintegration of a hyaluronic acid (HA) and hyaluronidase (HAS) used in prostate cancer radiotherapy.

**Materials and methods:**

Five in-vitro models are applicated with 3 ml (ml) HA. For dissolution varying doses of HAS were used: 6 ml, 3 ml, 1.5 ml, and 0 ml. One ml contains 150 International Units (IU). Each HAS was added with saline till the complementary amount of 6 ml. One phantom was solely implanted with a HA 3 ml acting as a control. Length, width and height were measured on different time points: 1st day 4 times, 2nd day 3 times, third day 2 times, and then once daily during one week, with a final measurement 2 weeks after implantation. The experiments were performed in duplicate to exclude variations and confirm the results.

**Results:**

The fastest dissolution was observed with the highest concentration of HAS, already observed at the first time point 2 h after implantation, with volume decrease of 50% on the second day, and less than 1 ml residue (33%) on day 4. The 2 other concentrations of HAS also showed a volume decrease, with less than 2 ml (66%) on day 4. All the applied quantities of HAS are observed with a residue of less than 1 ml after 7 days. After 14 days the control phantom and the saline filled one remains on steady state volume (3 ml).

**Conclusions:**

A dose response was observed by HAS injection: highest volumes of HAS dissolute most swiftly. Using a ratio of HA:HAS of 1:2 results in a decrease to half of initial volume within 24 h. This is of special interest when used in clinical practice following erroneous positioning, and dissolution is urgently needed.

## Highlights

• Hyaluronic acid (HA) is an implantable rectum spacer used to decrease rectal radiation dose in prostate cancer radiotherapy.
• Hyaluronidase (HAS) is an enzyme that degrades HA, when wrongly positioned.
• A ratio of HA:HAS of 1:2 has already a decrease of half of volume on the 2nd day.

## Introduction

Hyaluronic acid (HA) is one of the four commercialized implantable rectum spacers available on the market, besides hydrogel, saline filled balloon, and human collagen [1], [2], [3], [4]. These implantable rectum spacers are used to decrease rectal radiation dose in prostate cancer radiotherapy to avoid rectal complications after prostate cancer radiotherapy. Such side effects are common (up to 20%), especially during dose-escalation and hypofractionated radiotherapy [5], [6].

However implantation of such devices is not risk-free: implanting such spacers accidentally in the rectal wall, might result in a chronic wound. Then it can take 3 to 9 months for such spacers to resolve [7]. Furthermore, such a complication results in a significant treatment delay, since an immediate start of radiotherapy could increase the risk of fistula development. These grade ≥ 3 complications are luckily very rare in incidence (approximately 0.18% to 1.49%), however it should be completely avoided [7], [8]. Moreover if a spacer device is not satisfactory implanted in the correct position, a reposition would be of great interest. Therefore the feasibility to resolve such a spacer is of great interest in the community. A closed spacer system like the saline filled balloon can be easily punctured. On the other hand, for the liquid spacers (such as Hyaluronic acid, hydrogel, and human collagen) no clear solution is available. Until today, no knowledge is available on the dissolution of the HA used in prostate cancer radiotherapy in perspective of the timing and the required quantities of hyaluronidase (HAS), which is an enzyme that degrades HA. The use of HAS is widely employed in aesthetic medicine, due to their role in preventing complications from inappropriate injection of HA, like eliminating HA nodules, or correcting unsightly HA overfilling, which is labeled as safe [9]. Therefore this in-vitro analysis was performed to determine a dose response relationship of disintegration between HA and HAS.

## Materials and methods

This is an in-vitro study using the Simulated Inanimate Models of prostate (SIM^TM^, LLC, Rochester, NY, USA), while injecting hyaluronic acid (Barrigel®, Non-Animal Stabilised Hyaluronic Acid, NASHA®, Palette Life sciences, Stockholm, Sweden). Five models are used for implantation with 3 ml HA. For dissolution varying doses of clinical grade HAS (Mesomedica®, 150 International Units (IU)/ml) were used: 6 ml (900 IU), 3 ml (450 IU), 1.5 ml c (225 IU), and 0 ml, respectively. Each HAS was added with saline till the complementary amount of 6 ml: respectively 0 ml, 3 ml, 4.5 ml, and 6 ml saline is added. One phantom was solely implanted with a HA 3 ml acting as a control. Length, width and height were measured at the following time points: 2 h, 4 h, 6 h, 8 h after implantation, 2nd day 3 timepoints with an interval of 3 h, third day 2 times with interval of 4 h, then daily for one week, with a final measurement 2 weeks after implantation.

The experiments were performed in duplicate to exclude variation in measurements and variable effect of the used concentrations.

All dimensions of the gel were measured in millimeters (mm). These dimensions were measured with a bi-plane Trans Rectal Ultra sound probe (Pro Focus 2202 - BK Medical; transducer type 8848) using an ultrasound contrast gel-filled condom to improve visibility on the phantom, the prostate and rectum model, and the gel. On axial view the width and height are measured, and on sagittal view the length was detected. An absolute volume was calculated by using an ellipse volume formula [10]:

Volume gel (ml) = (length × width × height) × ϖ/6.

Relative volumes were calculated as the ratio of the measured volume of a certain time point to the measured volume of the first time point.

## Results

**Fig. 1:**
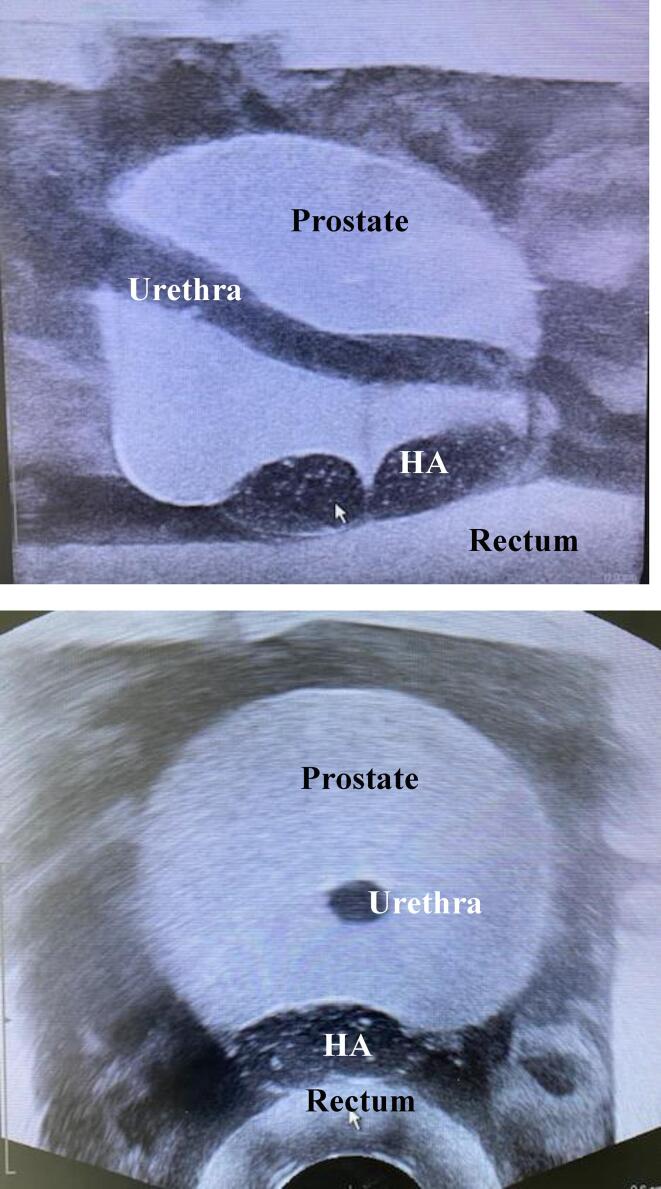
Axial and Sagittal ultrasound image of prostate SIM model with a Hyaluronic acid (HA) injection between the prostate and the rectum. Note the urethra is in the central plane: in the sagittal view the complete urethra is visible.

All HA were positioned and injected in the phantoms, i.e. between the prostate and the rectum model (Fig. 1).

**Fig. 2:**
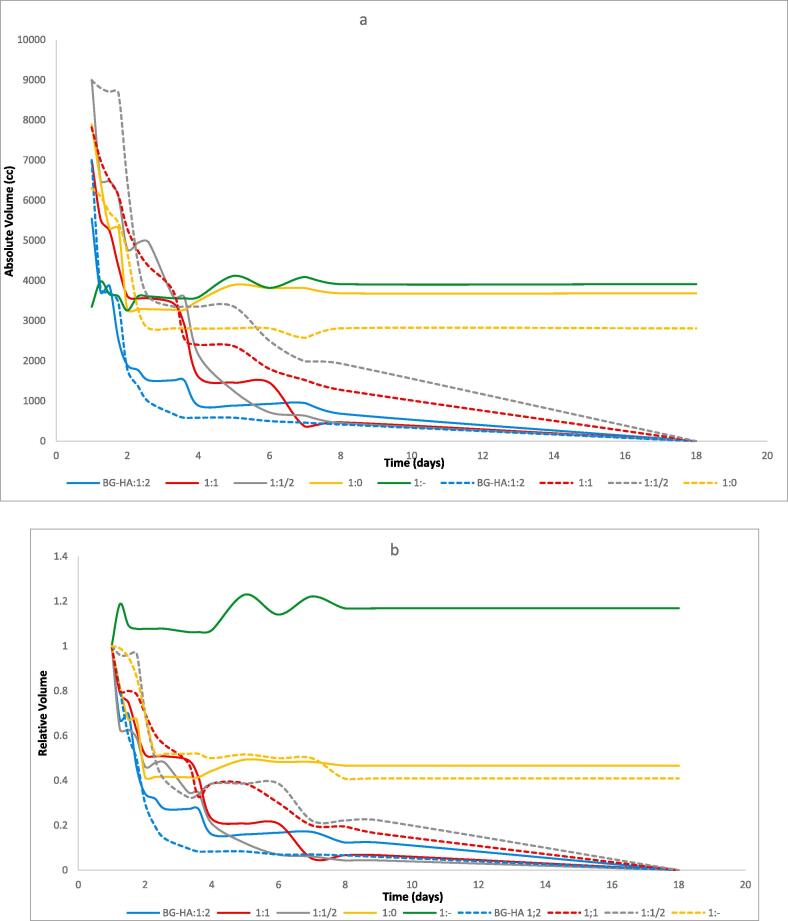
Absolute volume (a) and Relative volume (b) dynamics of HA implants during 2 weeks in 5 phantoms. The 2nd test is projected in dashed lines. There are 2 groups without HAS: one HA injection without HAS (the green line) and one HA injection with solely 6 ml saline (orange lines). The light blue line is the HA with HAS ratio 1:2 (3 ml HA with respectively 6 ml HAS). The red and grey lines are respectively the HA with HAS ratio of 1:1, and 1:1/2. (For interpretation of the references to colour in this figure legend, the reader is referred to the web version of this article.)

The absolute and relative HA volumes during different time points are presented in Fig. 2. The dissolution started immediately on day 1, with all forms of HAS. The most dramatic changes occurred during the first 2 days after HAS injection, with continued gradual degradation up to day 7, where only some residue was noted.

Dose responses of HAS were observed: the 900 IU/ml revealed already a decrease of half of volume on the second day, and less than 1 ml (33%) residue on day 4.

The differences in response decreased when the doses of HAS decreased. The 450 IU/ml and 225/ml of HAS were also decreasing relatively quickly, with less than 2 ml residue (66%) on day 4.

The duplicate experiments are presented in Fig. 2 as dotted lines, with a same trend as the first experiment.

At 2 weeks a steady state volume was observed for the HA control and saline injected HA.

## Discussion

This is the first study to examine and evaluate the volumes of HA gel dissolution with insertion of HAS in an in-vitro prostate cancer based on daily ultrasound measurements.

HA is a high-viscosity hydrophilic polysaccharide (glycosaminoglycan-based polymer) compound found in human tissues (in the skin, the synovial fluid of the joints) as a component of the connective and extracellular matrix. It has the capacity to hold water, which allows for reliable, reproducible injections which is of great interest in rectal spacing in prostate cancer radiotherapy to decrease radiation dose to the rectum. Despite the relatively high safety profile, several complications of HA fillers have been reported in other clinical setting like esthetic surgery, including granulomatous reactions, lumpiness, and skin necrosis [11].

In prostate cancer, the use of HA is described in only 2 small series [1], [12]. Prada et al. published on the use of the HA in combination with brachytherapy, and showed a relative reduction of 28% in the maximum rectum dose averaged over all patients in the cohort [1]. Both series reported no grade 3 side-effects. However complications with comparable liquid spacers (hydrogel) are reported, including prostatic abscess and sepsis, urinary retention, local rectal injury with rectal wall erosion, and rectourethral fistula [7]. The HA compound is cleaved by the enzyme HAS to its component subunits, which are predominantly eliminated by hepatic and renal metabolism [13]. Therefore the use of HAS is of great interest.

HAS is a successful rescue medicine for various complications of wrong HA injections. It has been approved for clinical use and is used to increase the HA diffusion into the extracellular matrix. In this study, we examined the timing of dissolution of HA following HAS insertion as a function of HAS concentration. A dose response was observed: the highest volumes of HAS (ratio HA-HAS 1:2) reached more than half of dissolution on day 2. This is of special interest when the HA is inserted accidentally in the rectal wall, and could give pressure on the rectum with consequently wound problems and erosions, with possible fistula formation. So the use of HAS in combination with HA can decrease this potential toxicity level of this specific liquid spacer. Lower doses of HAS are advised to insert when position is not satisfactory but when no pressure on critical organs is observed. The ratio HA-HAS 1:0 revealed after day 2 a steady state, which represents that the saline is resolved in the first days, however the HA remains in situ.

This study has some limitations. Firstly, it is an in-vitro study, so no body temperature is achieved, which could influence the disintegration process. When the pharmacokinetics and pharmacodynamics are observed in-vivo the situation is slightly different: the clinical effect is immediately with a reduction of swelling within minutes after administration [14]. A similar early effect was observed in the present setup, i.e. at 2 h, for the highest HAS concentration. The clearance of HAS in the serum occurs with a t½ of 2.1 ± 0.2 min, and is followed by inactivation in the kidneys and liver [15]. The duration of actions are regular reported as 24 to 48 h. So, the disintegration process in-vivo is probably much faster, and the required quantities of HAS could be lower. However, Casabona and co-authors described already different amounts (even up to 3-fold) of HAS necessary to degrade a same volume of HA [14].

Secondly, the measured volumes of complex structures are simplified as an ellipse, and are all based on solely 3 measurements (the width, height, and the length). This can give inaccuracies and uncertainties in the measured volumes, however the uncertainties are mainly systematic in each separate spacer implant, which makes the relevant volumes as most predominant to interpret.

Further, several HAS products demonstrated different activities because the extent of crosslinking and the molecular weight of HA of each product have a significant effect on the resistance to degradation.

Next, additional factors are influencing the interaction of HA and HAS, including, location of injection, and injection techniques [11].

Although this was an in-vitro study, the authors foresee that this quantitative analysis of HAS concentration is supportive for HAS injection to correct or dissolve HA rectum spacer injection errors which enables the continuation of the prostate cancer radiotherapy treatment, without delay.

## Conclusions

All volumes of hyaluronidase implantation resulted in a dissolution of HA with temporal differences observed between different concentrations. A dose response was observed: with the highest volumes of hyaluronidase most swiftly dissolution was reached. A ratio of 1:2 (3 ml HA with respectively 6 ml HAS, 900 IU) has a dissolution of more than half of volume on the second day. This is of special interest while using the hyaluronic acid in clinical practice in prostate cancer radiotherapy when wrongly positioned, and dissolution are urgently needed. If no urgency is required, a ratio of 1:½ is already enough to obtain a satisfied dissolution.

## Conflict of interest statement

All authors declare to have no conflict of interest.
